# The efficacy of intranasal leptin for opioid-induced respiratory depression depends on sex and obesity state

**DOI:** 10.3389/fphys.2023.1320151

**Published:** 2023-12-13

**Authors:** Michele L. Singer, Mi-Kyung Shin, Lenise J. Kim, Carla Freire, O Aung, Huy Pho, Joshua A. East, Frank P. Sgambati, Alban Latremoliere, Luu V. Pham, Vsevolod Y. Polotsky

**Affiliations:** ^1^ Division of Pulmonary and Critical Care Medicine, Department of Medicine, Johns Hopkins University School of Medicine, Baltimore, MD, United States; ^2^ The Johns Hopkins Center for Interdisciplinary Sleep Research and Education (CISRE), Johns Hopkins University School of Medicine, Baltimore, MD, United States; ^3^ Departments of Neurosurgery and Neuroscience, Johns Hopkins University School of Medicine, Baltimore, MD, United States, United States; ^4^ Department of Anesthesiology and Critical Care Medicine, George Washington University School of Medicine and Health Sciences, Washington, DC, WA, United States; ^5^ Department of Pharmacology and Physiology, The George Washington University School of Medicine and Health Sciences, Washington, DC, WA, United States

**Keywords:** opioids, respiration, leptin, obesity, apnea

## Abstract

**Introduction:** Opioid-induced respiratory depression (OIRD) is the primary cause of death associated with opioids and individuals with obesity are particularly susceptible due to comorbid obstructive sleep apnea (OSA). Repeated exposure to opioids, as in the case of pain management, results in diminished therapeutic effect and/or the need for higher doses to maintain the same effect. With limited means to address the negative impact of repeated exposure it is critical to develop drugs that prevent deaths induced by opioids without reducing beneficial analgesia.

**Methods:** We hypothesized that OIRD as a result of chronic opioid use can be attenuated by administration of IN leptin while also maintaining analgesia in both lean mice and mice with diet-induced obesity (DIO) of both sexes. To test this hypothesis, an opioid tolerance protocol was developed and a model of OIRD in mice chronically receiving morphine and tolerant to morphine analgesia was established. Subsequently, breathing was recorded by barometric plethysmography in four experimental groups: obese male, obese female, lean male, and lean female following acute administration of IN leptin. Respiratory data were complemented with measures of arterial blood gas. Operant behavioral assays were used to determine the impact of IN leptin on the analgesic efficacy of morphine.

**Results:** Acute administration of IN leptin significantly attenuated OIRD in DIO male mice decreasing the apnea index by 58.9% and apnea time by 60.1%. In lean mice leptin was ineffective. Blood gas measures confirmed the effectiveness of IN leptin for preventing respiratory acidosis in DIO male mice. However, IN leptin was not effective in lean mice of both sexes and appeared to exacerbate acid-base disturbances in DIO female mice. Additionally, morphine caused a complete loss of temperature aversion which was not reduced by intranasal leptin indicating IN leptin does not decrease morphine analgesia.

**Discussion:** IN leptin effectively treated OIRD in morphine-tolerant DIO male mice without impacting analgesia. In contrast, IN leptin had no effect in lean mice of either sex or DIO female mice. The arterial blood gas data were consistent with ventilatory findings showing that IN leptin reversed morphine-induced respiratory acidosis only in DIO male mice but not in other mouse groups. Finally, a hypercapnic sensitivity study revealed that IN leptin rescued minute ventilation under hypercapnic conditions only in DIO male mice, which suggests that differential responses to IN leptin are attributable to different leptin sensitivities depending on sex and the obesity status.

## 1 Introduction

Opioid addiction and misuse is a serious national crisis that affects public health, as well as social and economic welfare (Abuse, N.I. on D., 2023). The opioid crisis has been greatly exacerbated by the increased availability of highly potent synthetic opioids, such as fentanyl, and by the increased prescription of opioid pain relievers (Van Zee, 2009; Morone and Weiner, 2013). Opioid overdose kills 130 people in the United States every day, according to the CDC/NCHS National Vital Statistics System (“CDC WONDER,” 2023). The primary cause of death associated with opioids is opioid-induced respiratory depression (OIRD). A driving risk factor of OIRD is obesity, greatly increasing opioid-related mortality (Masters et al., 2018), which is mostly related to co-morbid sleep-disordered breathing (SDB) (Catley et al., 1985; Gross et al., 2006; Gali, 2009; Adesanya et al., 2010; Lam et al., 2016; Subramani et al., 2017). According to CDC, there were 43.3 opioid prescriptions per 100 people in the United States in 2020, and many patients requiring high doses of opioids are habituated to chronic opioid intake (Madadi et al., 2013).

Opioids act on μ-opioid receptors (MORs) within the brainstem, including the pre-Bőtzinger complex (preBőtz) and the ventral respiratory group, to suppress the respiratory drive and decrease the respiratory rate (Smith et al., 1991; Gray et al., 2001; Montandon et al., 2016; 2011; Montandon and Horner, 2014). Naloxone is a competitive antagonist of MORs and other opioid receptors (Pasternak and Pan, 2013; Wong et al., 2017). It has a rapid onset, and it is highly effective in reversing OIRD, but it is short-acting, induces acute withdrawal, and counteracts opioid analgesia. There is no FDA-approved OIRD treatment that maintains opioid analgesia.

We have used our diet-induced obesity (DIO) mouse model of SDB and have shown that intranasal (IN) leptin treats OIRD in DIO male mice, decreasing the number of apneas and increasing minute ventilation without decreasing analgesia (Freire et al., 2020). However, leptin’s efficacy in female and in non-obese subjects has not been tested. The main goal of our study was to determine whether IN leptin is effective for OIRD treatment in opioid-tolerant subjects of both sexes in the absence and presence of obesity.

We have used our mouse morphine model of OIRD, in which IN leptin showed high efficacy (Freire et al., 2020), and designed a series of experiments in lean and DIO C57BL/6J male and female mice to address this aim. First, we established a protocol of morphine tolerance using chronic subcutaneous morphine infusion. Second, we determined a dose of morphine which would induce OIRD in morphine-tolerant mice. Third, we performed IN leptin dose titration by measuring breathing in morphine-treated mice. Fourth, we measured the hypercapnic ventilatory response in morphine-treated mice with or without IN leptin. Fifth, we measured arterial blood gas in unanesthetized unrestrained morphine-treated mice with or without IN leptin.

## 2 Methods

### 2.1 Animals

In total, 119 male and 89 female C57BL/6J mice, aged 20–25 weeks, were utilized across all experiments (Jackson Laboratories, Bar Harbor, ME). The mature adult age range includes ages from 3 to 6 months, and the variation in mice ages in these experiments is related to the differences in the rate of weight gain required to achieve the “obese” status. The target weights for DIO male mice were >45 g and for DIO female mice >40 g. Mice were utilized in experiments as follows: 1) establishment of a model of tolerance to the analgesic effects of morphine (DIO and lean mice, *n* = 24); 2) dynamic thermal place preference protocol (DIO mice, *n* = 8); 3) establishment of a model of OIRD in morphine-tolerant mice (DIO and lean mice, *n* = 8); 4) apnea dose response in morphine-tolerant mice (DIO and lean mice, *n* = 50); 5) measurement of the effect of acute administration of IN leptin on OIRD in mice chronically receiving morphine and tolerant to morphine analgesia (DIO and lean mice, *n* = 38); 6) measurement of the effect of IN leptin on arterial blood gas in morphine-treated mice (DIO and lean mice, *n* = 80). Water was provided to all animals *ad libitum*. Eighty seven mice (51 males and 36 females) were provided standard chow diet *ad libitum* except during sleep recording. In total, 121 mice (68 males and 53 females) were provided high-fat diet *ad libitum* (60% of kcal from fat, D12492, research diets) from the time of arrival in the animal facility through the end of experimental procedures, except during sleep recording. Mice were housed in a standard laboratory environment at 24°C–26°C in a 12-h light–dark cycle (9 a.m.–9 p.m. lights on). The study was approved by the Johns Hopkins University Animal Use and Care Committee (Protocol: MO21M165) and complied with the American Physiological Society Guidelines for Animal Studies.

### 2.2 The Establishment of a model of tolerance to analgesic effects of morphine in mice

**FIGURE 1:**
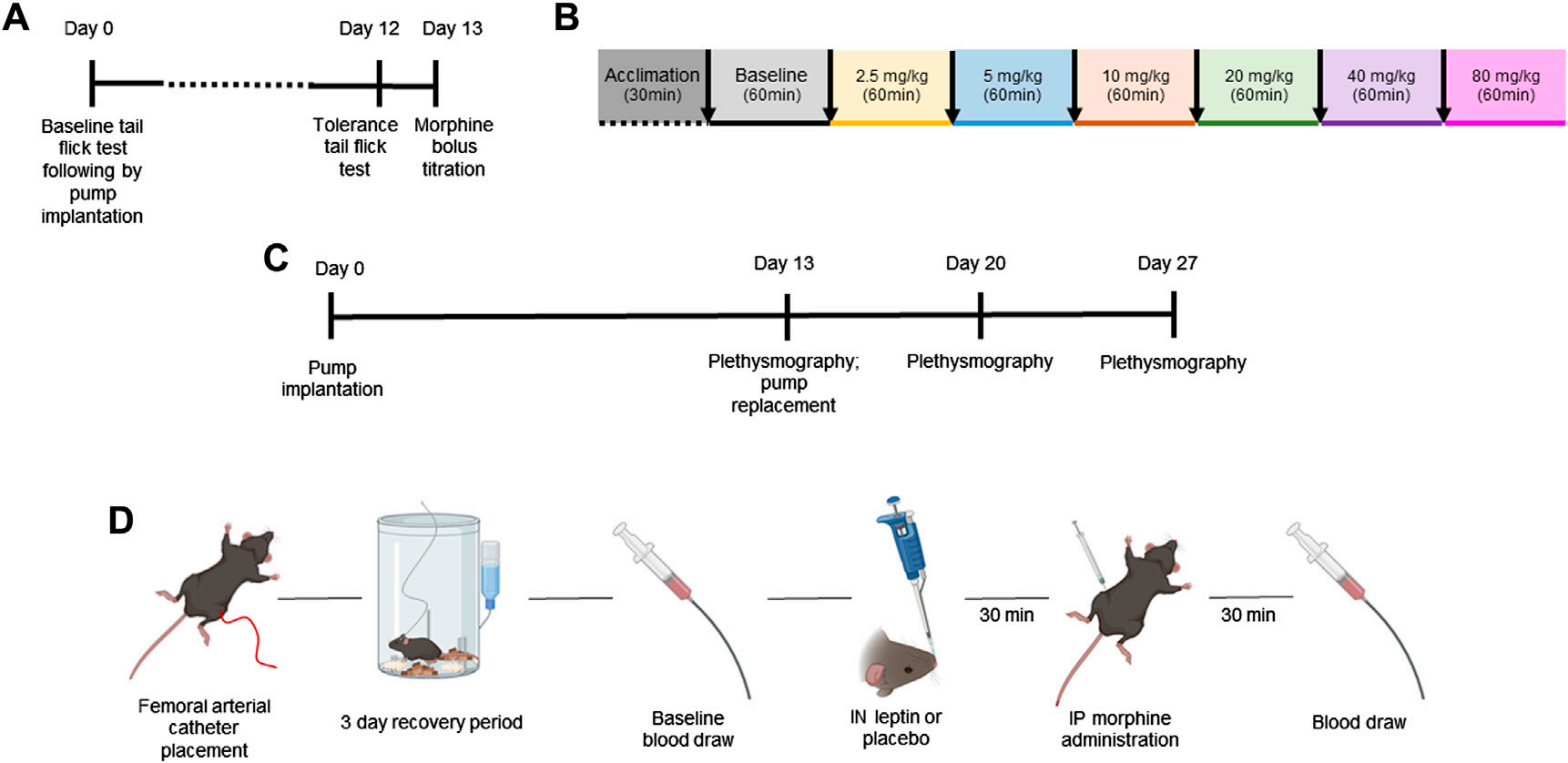
Study design. Mature adult mice aged 20–25 weeks of age were used for all experiments. The aim of the first set of experiments was to establish a model of tolerance to the analgesic effects of morphine in mice **(A)**. DIO and lean mice (*n* = 12 per group) were randomly assigned to receive a subcutaneous pump (Alzet) delivering morphine at 7.5 mg/kg/day or 12 mg/kg/day, and the tail flick test was performed at baseline and on day 12 after pump implantation. On day 13, a model of OIRD was established in morphine-tolerant mice (DIO *n* = 5; lean *n* = 3). Respiratory recordings were performed in a plethysmography chamber on day 13 after pump implantation at baseline and after morphine dose escalation every 60 min **(B)**. **(C)** Design of a separate experiment in which morphine-tolerant mice received morphine bolus titrated in experiments **(A, B)** with IN leptin (dose range from 0 to 4.8 mg/kg) administered 30 min prior to morphine bolus on days 13, 20, and 27, following morphine pump implantation. **(D)** Finally, blood gas experiments were conducted to compliment respiratory data. Mice were implanted with the femoral arterial catheter, followed by a 3-day recovery period. On the day of testing, baseline blood was collected immediately followed by IN leptin or placebo administration. Then, 30 min later, mice received IP morphine, and 30 min after the morphine injection, final blood gas samples were taken for analyses (*n* = 57).

DIO and lean mice (*n* = 12 per group) were randomly assigned to receive a subcutaneous pump (Alzet, model 2002) delivering daily doses of 7.5 mg/kg/day or 12 mg/kg/day morphine (MWI Animal Health, Boise, ID) at a rate of 0.5 μL/h. For pump implantation, mice were anesthetized with 1%–2% isoflurane, and a small incision was made with a scalpel in the skin of the left flank of the mouse. A small subcutaneous pocket was formed using forceps, a pump was inserted, and the surgical wound was closed with a suture (Kliewer et al., 2019). The tail flick test was performed as previously described (Wang et al., 2009; Glovak et al., 2017) at baseline and on day 12 after pump implantation (Figure 1A). In brief, mice were acclimated to a restrainer tube for 3 days, immobilized in an acrylic tube, and their distal tail was immersed in water at 50°C ± 1°C. The latency was recorded the moment a tail flick was observed. A maximum of 15 s immersion time was allowed to avoid tissue damage. After baseline measures, mice received IP morphine (10 mg/kg) or placebo. Tail flick latencies were measured at baseline, 15, 30, 60, and 120 min after IP morphine/placebo administration. Tolerance to analgesic effects of morphine was expressed as reduction in the maximal possible effect (MPE) after 12 days of morphine infusion.

### 2.3 Dynamic thermal place preference protocol

Two thermal plates were placed directly adjacent to one another with a plastic chamber surrounding both, allowing for free movement between each plate (Bioseb, France). Each plate started at 30°C; then, one plate increased the temperature to 50°C over a 200-s period. When the first plate reached 50°C, it started cooling down to 30°C, while the second plate started increasing its temperature to 50°C. The gradient of temperature change was equal on both plates but opposite, ensuring that as one plate’s temperature increased toward 50°C, the alternate plate’s temperature reduced toward 30°C. At any given time, one of the plates was always at an innocuous temperature to allow the mouse to escape from the hot plate. Each experimental run lasted 20 min and provided three or four exposures to increasing temperature. The time spent on each plate was automatically recorded, and the threshold to leave the hot plate was calculated. A transition was defined as when the animal spent more than at least 10 s on a different plate. Animals were tested for three baseline runs, followed by either morphine (10 mg/kg) + intranasal (IN) vehicle or morphine (10 mg/kg) + IN leptin. Half of the mice were exposed to morphine + leptin first, followed by morphine + vehicle, and the other half had the mirror dosage protocol. Brief exposure to isoflurane (2 min at 3%) or IN leptin alone did not change place aversion readouts. The experiments were performed in at least two independent sets of mice.

### 2.4 Establishment of a model of OIRD in morphine-tolerant mice

On day 13 after morphine pump implantation (12 mg/kg), morphine bolus was delivered via intraperitoneal injection with mice (DIO male mice *n* = 5; lean male mice *n* = 3) placed in the head-down position. A 1.0-mL insulin syringe was used to inject the morphine solution into the lower right quadrant of the abdomen. Increasing doses of morphine (0, 2.5, 5, 10, 20, 40, and 80 mg/kg) were administered to DIO and lean male mice with a 60-min interval between injections (Figure 1B). Respiratory recordings were performed in a plethysmography chamber, and apneas were scored when the flow was absent for a period corresponding to two or more breaths. The results were expressed as % of apnea at baseline (chronic morphine infusion without morphine bolus).

### 2.5 Effect of acute IN leptin on OIRD

To determine the efficacy of IN leptin in OIRD in morphine-tolerant mice (20–22 weeks of age), an apnea dose–response curve to leptin (R&D Systems, Inc., Minneapolis, MN) was performed in 50 mice: DIO male (*n* = 12), DIO female (*n* = 22), lean male (*n* = 8), and female mice (*n* = 8). In all mice included in the OIRD protocol, the subcutaneous morphine pump was implanted as described above, delivering morphine at a rate of 12 mg/kg/day based on experiments described in Section 2.2. On day 13, mice were randomized and then treated with IN leptin at doses of either 0 (placebo, saline, Thermo Fisher Scientific Inc., United States), 0.6, 1.2, 2.4, and 4.8 mg/kg, followed by an IP morphine bolus at 5 mg/kg (in lean mice) or 10 mg/kg in DIO mice, followed by the plethysmography recording. The morphine bolus dose was determined based on the experiments explained in Section 2.4. Airflow and respiratory efforts were recorded for 2 h after morphine injection, and a number of apneas per hour were determined. Each mouse received three different doses of leptin + IP morphine bolus 1 week apart (days 13, 20, and 27, Figure 1C), while morphine infusion continued. A higher number of DIO female mice were used as female subjects are prone to significant weight loss, following implantation of the opioid pump. Specifically, two female mice stopped eating and drinking prior to the first dose of leptin and were euthanized. Eight female mice appeared ill, disheveled, and demonstrated a rapid weight loss of 15%–33% at which time they were excluded from additional testing. In all, 10 female DIO mice were excluded.

### 2.6 Respiratory measures

In total, 38 mice were used for respiratory measurements (DIO males *n* = 11; DIO females *n* = 11; lean males *n* = 8; and lean females *n* = 8). A two-arm crossover study design was used, and mice were randomized to receive leptin or placebo via IN injection. After a 1-week washout period, each mouse underwent testing again under the opposite condition. A whole-body plethysmography (WBP) chamber system was used to measure the tidal airflow, generating high-fidelity tidal volume and airflow signals as previously described (Hernandez et al., 2012). In brief, mice were weighed and subsequently placed in the WBP chamber for recording from 10:00 a.m. to 4:00 p.m. The mouse rectal temperature was measured and averaged between the beginning and end of the study. The reference chamber of the WBP filtered out ambient noise from the pressure signal acquired by a transducer (emka Technologies), and positive and negative pressure sources were utilized in series with mass flow controllers (Alicat Scientific) and high-resistance elements to generate a continuous bias airflow through the chamber while maintaining a sufficiently high time constant. Using the pressure signal from the WBP chamber, the tidal airflow was calculated by applying the Drorbaugh and Fenn equation (Drorbaugh and Fenn, 1955), which required measurements of the mouse rectal temperature, chamber temperature, relative humidity, room temperature, and chamber gas constant calculated by using a known volume injection and the resultant chamber deflection. The tidal volume signal was differentiated electronically to generate an airflow signal. Signals were digitized at 1,000 Hz (sampling frequency per channel) and recorded in LabChart Pro (Version 7.4, ADInstruments, Dunedin, NZ). Mice were acclimated to the WBP chamber for a minimum of 3 hours approximately 1 week prior to experimental recording. For analysis, we performed breath detection, as previously described. In brief, stretches of ∼30 s were manually sampled every 300 s. The respiratory rate was measured, tidal volume (V_T_) was calculated by integrating the flow during each inspiration, and V_E_ was normalized by body weight. Analyses were performed in R version 4.2.2. Hypercapnic sensitivity was analyzed by exposure to 8% CO_2_ balanced in room air. Mice were exposed to three cycles of hypercapnia (8% CO_2_ at 20.9% FiO_2_ balanced in N_2_) of 5 min/each. CO_2_ exposures were alternated with 20 min of recordings under room air conditions**.**

### 2.7 Blood gas measures

In total, 80 mice (*n* = 20 in each group: DIO male, DIO female, lean male, and lean female mice) underwent left femoral artery cannulation under 1%–2% isoflurane anesthesia, as previously described (Fleury Curado et al., 2018; Caballero-Eraso et al., 2019). In brief, a small incision was made in the inguinal area along the natural angle of the hind leg to expose the femoral artery, and an arterial catheter was placed 5–8 mm into the femoral artery. The catheter was glued into place, and the opposite end of the catheter was fed subcutaneously to an incision in the neck for dorsal attachment to a single channel fluid swivel (model 375/25, Instech Laboratories, Plymouth Meeting, PA, United States), which slowly perfused a heparin–saline solution (1,000 U heparin/L saline) via an infusion pump (0.5 mL/day). Mice recovered for 72 h and arterial blood samples (∼95 µL) were acquired for analyses in awake, unrestrained, unanesthetized mice via a femoral arterial line. Mice were randomized to receive IN leptin (1.2 mg/kg, ∼24 µL) or placebo (saline carrier, ∼24 µL) immediately after the baseline blood sample was obtained and analyzed. Then, 30 min after IN injection, mice were given a bolus of morphine (10 mg/kg) via intraperitoneal injection with a final blood gas sample collected 30 min after morphine injection Figure 1D. Blood samples for each mouse were tested in a blood gas analyzer (i-STAT1 analyzer, Abbott, United States). Initial blood gas experiments were attempted in morphine-tolerant mice. However, mice demonstrated severe akathisia (incessant movement) following morphine pump placement which resulted in twisting and disloding of the femoral arterial catheters. Although blood collection in morphine-tolerant animals was attempted, persistent issues with the femoral arterial catheters prevented consistent blood sampling, so all subsequent blood gas experiments were conducted in morphine-naïve animals. In addition, several animals (23 of 80) were excluded from final analysis. The high attrition rate is due to the fragile nature of the surgical placement of the femoral arterial line and the extended recovery period (≥3 days) required with the femoral artery catheter in place. Mice were provided IP hydration and pain medication daily, as well as heparinized saline via the femoral line post-operatively. However, complications such as death prior to the experimental time point (e.g., dislodged catheter resulting in excessive blood loss) or technical issues (e.g., decreased patency of the femoral arterial catheter, resulting in an insufficient blood sample) required exclusion of mice from all groups. The final experimental numbers are as follows: DIO males *n* = 16, DIO females *n* = 14, lean males *n* = 12, and lean females *n* = 15.

### 2.8 Statistical analysis

The effects of increasing doses of morphine, as well as the effect of morphine + leptin on pain tolerance (tail flick), and respiratory measures were analyzed within the same mouse under different conditions in a crossover manner. The variances of the effects of the treatments on each parameter at each time point were assessed by repeated measures analysis using a univariate linear model. The variances of the differences within subjects in the repeated measures were evaluated by Mauchly’s sphericity test, which considered data spherical when *p* = 0.05. When data did not meet sphericity requirement, F values, effect size, and observed power were corrected using the Greenhouse–Geisser correction. For thermal place preference testing, a two-way ANOVA was used, followed by Tukey’s multiple comparison test. When assessing leptin dosing, mice were not tested across all of the range of leptin doses. Therefore, statistical analysis was performed using the Kruskal–Wallis and Mann–Whitney tests with paired t-tests run on paired samples. Statistical analyses were conducted in SPSS version 20.0 (IBM SPSS Inc.). For analysis of blood gas outcomes, statistical analyses were completed using PRISM GraphPad statistical software (version 10.0.0 for Windows, GraphPad Software, Boston, Massachusetts, United States). Using the mixed linear model, statistical significance was established at the 0.05 level and adjusted for any violation of the assumption of sphericity in repeated measures using the Greenhouse–Geisser correction. Treatment (placebo, leptin) or weight (DIO, lean) and sex (male, female) were included as model variables, using animals as a random effect. When appropriate, *post hoc* analyses were conducted using the Tukey–Kramer honest significant difference (HSD) test. Normality of the distribution was assessed using the Shapiro–Wilk test within each animal.

## 3 Results

### 3.1 Morphine tolerance and apnea dose response in lean and DIO mice

**FIGURE 2:**
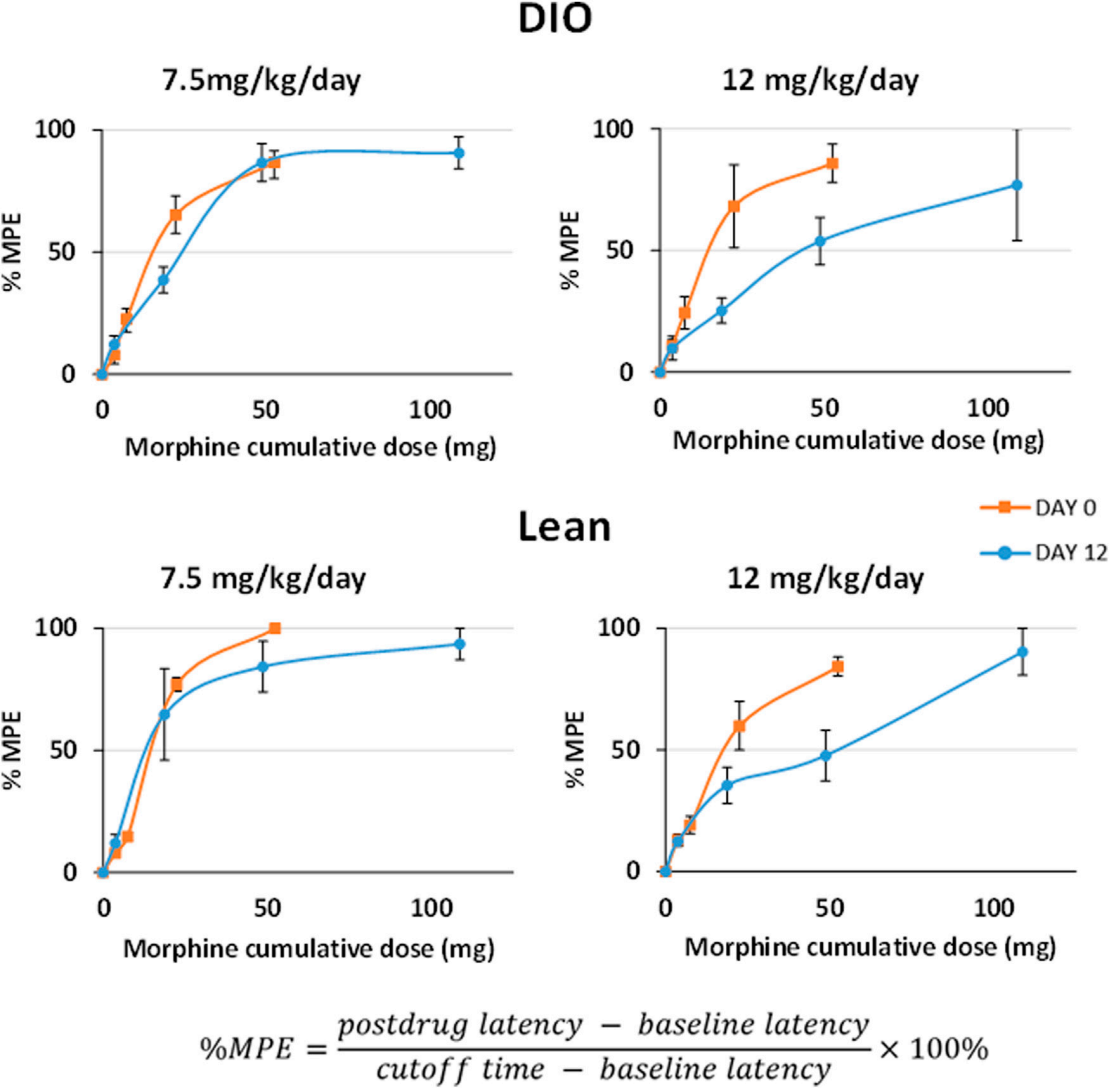
Development of morphine tolerance according to the tail flick test. Testing was performed prior to and 12 days after chronic subcutaneous morphine infusion at 7.5 and 12 mg/kg/day (*n* = 12 males per group). After baseline measures, the mice received IP morphine (10 mg/kg). Tail flick latencies were measured at baseline, 15, 30, 60, and 120 min after IP morphine administration. Tolerance to analgesic effects of morphine was expressed as reduction in the maximal possible effect (MPE) with a cutoff time of 15 s. Statistical analysis was completed within the same mouse under different conditions by means of a repeated measures analysis using a univariate linear model. Repeated measures were evaluated by Mauchly’s sphericity test, and when appropriate, corrections were made using the Greenhouse–Geisser correction.

**FIGURE 3:**
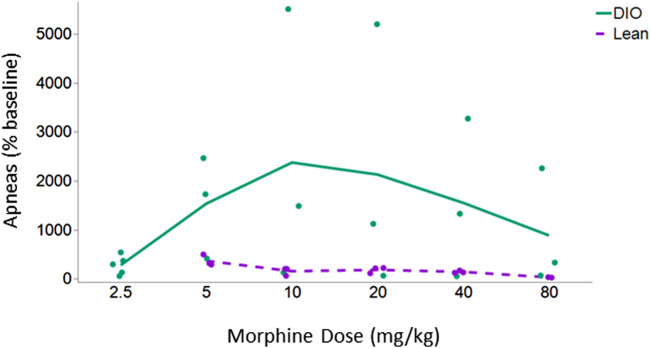
Apnea dose–response curve in lean and DIO morphine-tolerant mice. DIO morphine-tolerant male mice (*n* = 5) were more sensitive to the morphine bolus than lean male mice (*n* = 3) (*p* < 0.05). In subsequent experiments, we opted to use the dose of 10 mg/kg of morphine in DIO mice and 5 mg/kg in lean mice. Due to the significant variability between the mice at baseline, results are presented as % of baseline. The effect of increasing doses of morphine was analyzed within the same mouse under different conditions by means of a repeated measures analysis using a univariate linear model. Repeated measures were evaluated by Mauchly’s sphericity test, and when appropriate, corrections were made using the Greenhouse–Geisser correction.

The initial objective in this series of experiments was to determine the dose response to IN leptin required to counteract morphine-induced apnea in DIO and lean mice tolerant to morphine analgesia. For this purpose, we first established a model of tolerance in which morphine infusion at 12 mg/kg/day, but not 7.5 mg/kg/day, induces tolerance to the analgesic effects of the drug, as evidenced by response to the tail flick test (Figure 2). Therefore, morphine treatment at 12 mg/kg/day was used to induce tolerance to morphine analgesia in our experiments**.** Once morphine tolerance was established, the intraperitoneal dose of morphine bolus, which induces the greatest number of apneas (% baseline), was identified in respiratory recordings obtained in a plethysmography chamber. DIO morphine-tolerant mice were more sensitive to the morphine bolus than lean mice (*p* < 0.05). In lean mice, morphine at 5 mg/kg induced the greatest number of apneas per hour, whereas in DIO mice, it was 10 mg/kg (Figure 3). Based on these results, we opted to use the dose of 10 mg/kg of morphine in DIO mice and 5 mg/kg in lean mice for subsequent experiments.

### 3.2 The effect of intranasal leptin on morphine analgesia

**FIGURE 4:**
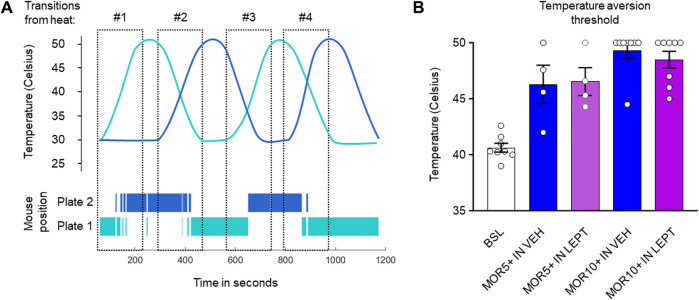
IN leptin does not diminish morphine analgesia, as measured by the thermal place preference (TPP) test. **(A)** Representation of the changing temperature of the thermal plates and the position of the mouse over time. **(B)** Temperature aversion thresholds in DIO male mice after IN leptin and morphine, as well as the IN vehicle and morphine. Each dot represents one animal (*n* = 5–8 per group). Data were analyzed by means of a two-way ANOVA, followed by Tukey’s multiple comparison test.

Previous experiments indicated that IN leptin does not diminish morphine analgesia measured in the tail flick test, a spinal reflexive assay. We performed additional testing using an operant assay: the dynamic thermal place aversion assay (Cobos et al., 2018). This operant assay represents a more integrated behavioral response to a noxious stimulus than purely reflexive assays. In untreated DIO male mice, the thermal aversion threshold was 40.6 ± 0.4C, similar to that of lean male mice. Morphine caused a dose-dependent loss of temperature aversion with a maximum effect at 10 mg/kg i.p. (49.3 ± 0.7C, *p* < 0.0001), and this was not changed by IN leptin (48.5 ± 0.8C, *p* < 0.0001) (Figure 4). Therefore, we conclude that IN leptin does not decrease morphine analgesia.

### 3.3 The Effect of acute administration of intranasal leptin on opioid-induced respiratory depression in mice chronically receiving morphine and tolerant to morphine analgesia

**FIGURE 5:**
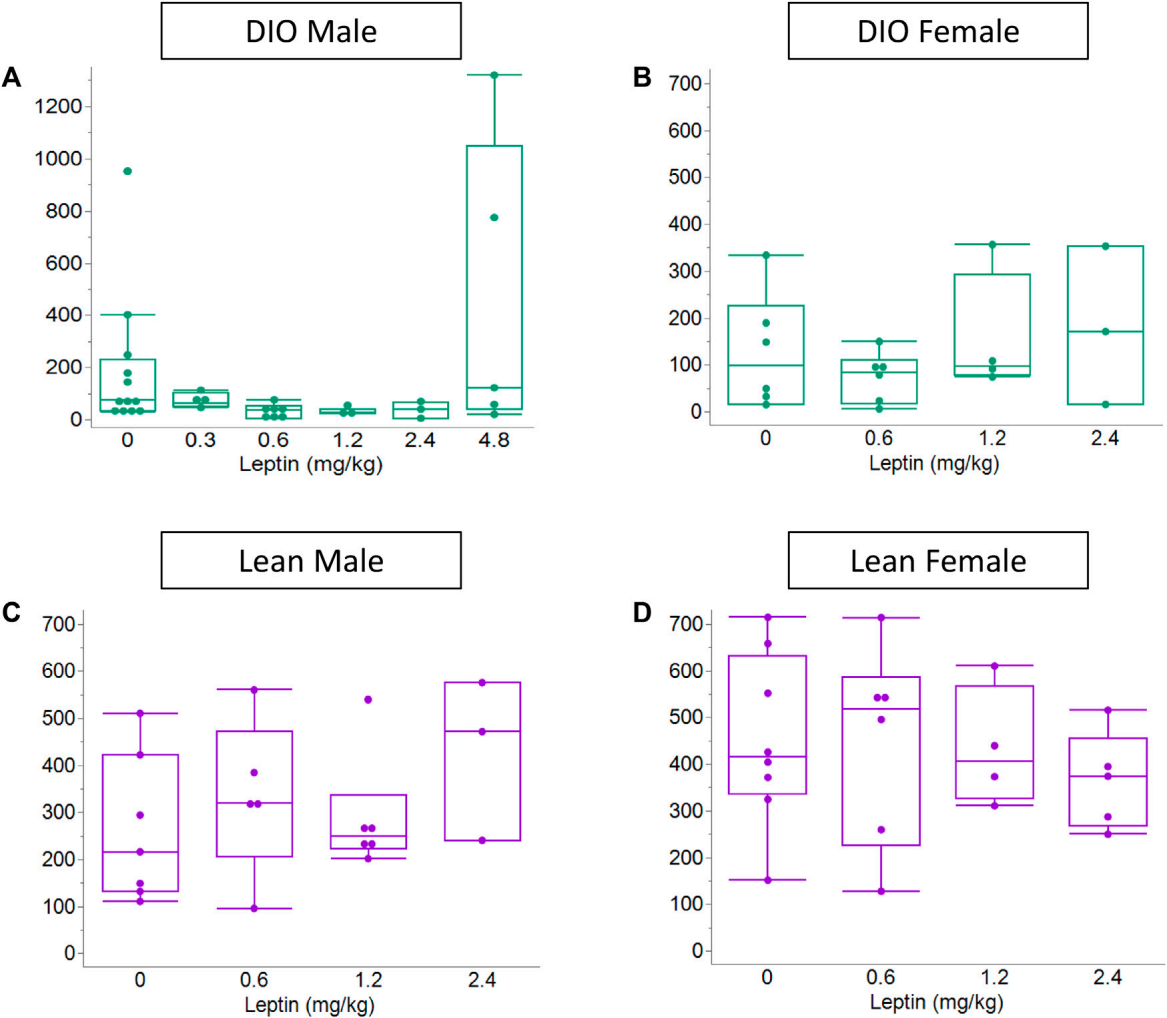
Effect of the acute administration of intranasal leptin on the number of morphine-induced apneas. On day 13, after pump implantation, airflow and respiratory efforts were recorded in plethysmography chambers. Data shown for all four experimental groups: **(A)** DIO male (*n* = 12), **(B)** DIO female (*n* = 12), **(C)** lean male (*n* = 8), and **(D)** lean female (*n* = 8). Acute administration of IN leptin substantially attenuated OIRD in DIO male mice, decreasing the apnea index by approximately 57%. However, the highest tested dose of IN leptin (4.8 mg/kg) appeared to increase apneas **(A)**. In DIO female and lean mice, leptin was ineffective **(B–D)**. Statistical analysis was performed using the Kruskal–Wallis and Mann–Whitney tests with paired t-tests run on paired samples.

In order to determine the efficacy of IN leptin in OIRD, we performed an apnea dose–response curve to leptin in DIO and lean mice (male and female). Of note, in the absence of leptin, both DIO and lean female mice showed significantly higher number of apneas in response to 10 mg/kg of morphine when compared to male mice (Figure 5). Additional information about sex differences is provided in Section 4. During the protocol, DIO male mice lost 3.5% ± 1.0% of body weight (from 46.3 ± 0.9 to 44.7 ± 1.0 g, *p* < 0.01). There was significant variability in apnea responses between mice (Figure 5). In DIO male mice, the median number of apneas per hour in the placebo group was 87.8. It was unchanged by IN leptin treatment at 0.3 mg/kg but decreased to 40.8, 33.9, and 50.7 after IN leptin at 0.6, 1.2, and 2.4 mg/kg (*p* < 0.05), respectively (Figure 5). In contrast, a highest tested dose of IN leptin, 4.8 mg/kg, appeared to increase apneas. Paired comparisons within the same animals confirmed that IN leptin at the lowest effective dose, 0.6 mg/kg, reduced the apnea index from 94.6 ± 29.8 to 40.9 ± 13.6 events per hour (57% reduction**,** *p* = 0.03). DIO female mice were tested at the final weight of 48 ± 0.6 g (*n* = 8). In DIO female mice, IN leptin at any concentration did not have an effect on the apnea index (Figure 5). Lean mice did not lose any weight during the experiment (from 30.3 ± 0.6 g to 30.0 ± 0.7 g in male mice and 23.3 ± 0.3 g to 24.5 ± 0.4 g in female mice). In contrast to the DIO male mice, IN leptin was ineffective in lean mice for OIRD prevention (Figure 5). We determined that an acute administration of IN leptin significantly attenuated OIRD in DIO male mice, decreasing the apnea index by almost 57%. In DIO female and lean mice of both sexes, leptin was ineffective. Although 0.6 mg/kg was found to be the lowest effective dose, the 1.2 mg/kg dose yielded similar results with less variability across mice. Therefore, 1.2 mg/kg dosing was used for subsequent experiments.

### 3.4 The effect of intranasal leptin on the hypercapnic ventilatory response in morphine-treated mice

**FIGURE 6:**
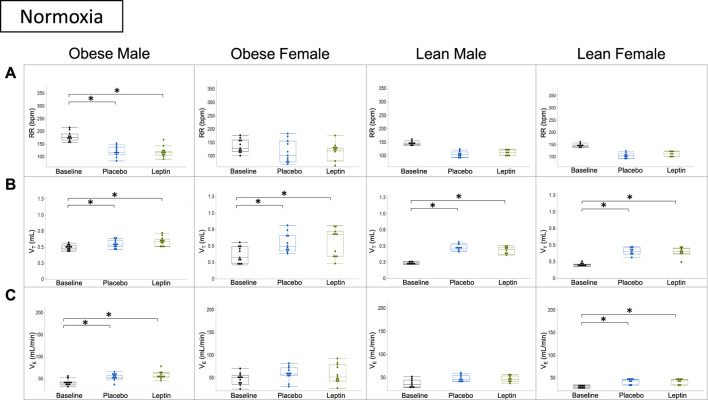
Effect of acute intranasal leptin on ventilation in morphine-treated mice. Respiratory rate **(A)**, tidal volume **(B)**, and minute ventilation **(C)** are shown for all four experimental groups (obese male *n* = 11; obese female *n* = 11; lean male *n* = 8; lean female *n* = 8). Under normoxic conditions (0% CO_2_), morphine suppresses the respiratory rate and increases minute ventilation in all groups except for obese female mice who appear to have preserved function, regardless of intervention. Tidal volume is the only measure in which all groups showed significant increase from baseline measures (*, *p* ≥ 0.05). Respiratory measures were analyzed within the same mouse under different conditions (i.e., normoxia vs. 8% CO_2_) in a cross-over manner. The variances of the effects of the treatments on each parameter at each time point were assessed by repeated measures analysis using a univariate linear model.

In subsequent experiments, we assessed the effect of leptin on hypercapnic sensitivity. Under normoxic conditions (0% CO_2_), morphine decreased the respiratory rate in DIO male and lean mice of both sexes, but there was no overall decrease in minute ventilation because of increases in the tidal volume. In contrast, morphine did not affect the respiratory rate or minute ventilation in DIO female mice. In DIO male mice, leptin did not block morphine-induced changes in breathing under normoxic conditions. Compared to morphine + placebo, IN leptin had no effect in any mouse group (Figure 6).

**FIGURE 7:**
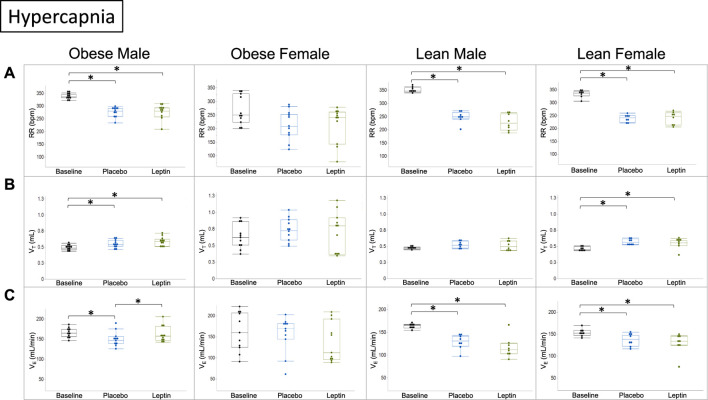
Effect of intranasal leptin on the hypercapnic ventilatory response in morphine-treated mice. Respiratory rate **(A)**, tidal volume **(B)**, and minute ventilation **(C)** are shown for all four experimental groups (obese male *n* = 11; obese female *n* = 11; lean male *n* = 8; lean female *n* = 8) under baseline, placebo, and leptin conditions during 8% CO_2_ challenge. Intranasal leptin had a protective effect against OIRD induced by morphine in obese male mice only, as demonstrated by an increase in minute ventilation. Obese females had preserved function across all measures, regardless of intervention. Intranasal leptin was ineffective in lean animals, in which leptin resistance is absent (*, *p* ≥ 0.05). Respiratory measures were analyzed, as described in Figure 6.

Under hypercapnic conditions (8% CO_2_), morphine decreased the respiratory rate and minute ventilation in DIO male mice despite increases in tidal volumes. IN leptin protected minute ventilation which remained at baseline and was significantly higher than in the placebo group. Lean mice of both sexes showed significant morphine-induced respiratory depression due to the suppressed respiratory rate and no effect of leptin. In DIO female mice, neither leptin nor morphine affected minute ventilation (Figure 7).

**FIGURE 8:**
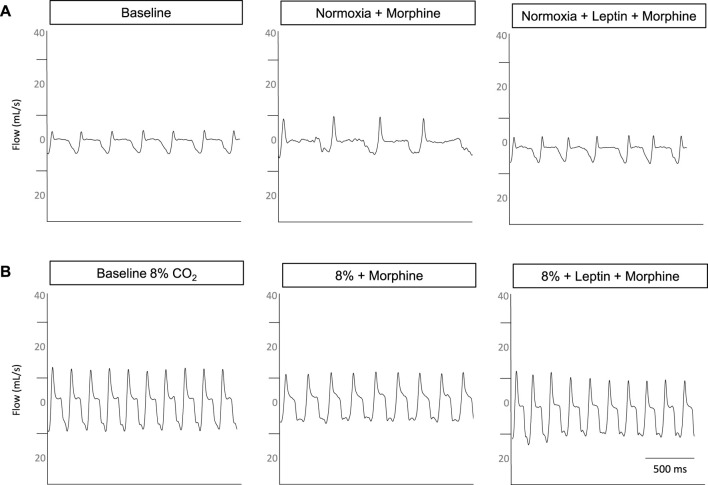
Representative respiratory traces from a DIO male mouse. Under normoxic conditions **(A)**, morphine increases the respiratory flow and decreases the frequency with the baseline respiratory pattern restored when intranasal leptin is administered. Under hypercapnic conditions **(B)**, flow is mildly suppressed and frequency is diminished, following morphine bolus; however, the administration of intranasal leptin appears to restore flow and frequency.

Representative respiratory traces in the DIO male mouse illustrate morphine-induced OIRD and leptin rescue during hypercapnic conditions (Figure 8), prompting further assessment with measures of arterial blood gas.

### 3.5 The effect of intranasal leptin on arterial blood gas in morphine-treated mice

**FIGURE 9:**
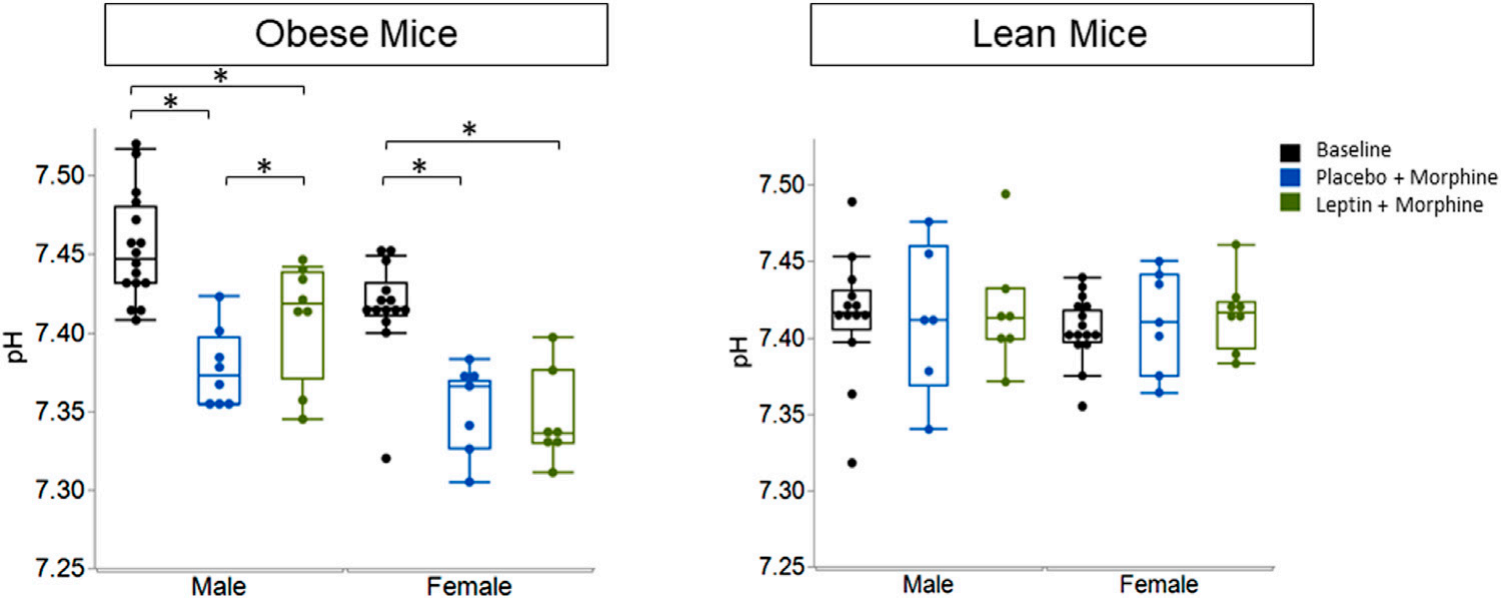
Blood gas: pH in obese and lean mice, following intraperitoneal injection of morphine. In previous studies, we have shown normal pH values in unanesthetized mice to be 7.46 ± 0.06. Here, we found that obese male mice treated with morphine + placebo had a pH of 7.38 ± 0.02, while those treated with leptin had significantly higher pH values which were closer to “normal” (7.41 ± 0.04). Leptin appears to be ineffective in lean male mice (placebo 7.41 ± 0.03; leptin 7.41 ± 0.02) and lean female mice (placebo 7.41 ± 0.05; leptin 7.42 ± 0.04) (obese male *n* = 16; obese female *n* = 14; lean male *n* = 12; obese female *n* = 15). Statistical analyses for all blood gas measures were conducted using a mixed linear model with the adjustment for sphericity in repeated measures using the Greenhouse–Geisser correction. When appropriate, *post hoc* analyses were completed by means of the Tukey–Kramer honest significant difference test.

**FIGURE 10:**
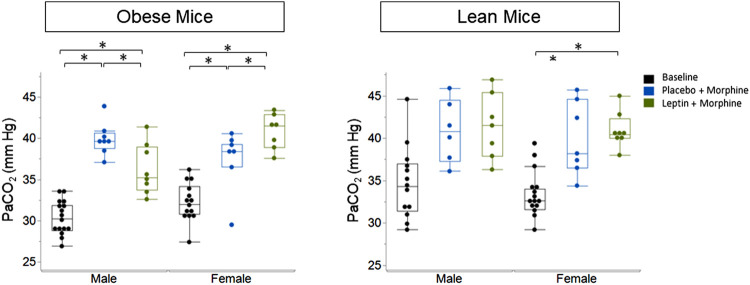
Blood gas: partial pressure of carbon dioxide (PaCO_2_) in obese and lean mice, following intraperitoneal injection of morphine. Obese male mice treated with an acute administration of intranasal leptin (36.25 ± 3.06) demonstrated a significant decline in PaCO_2_ (*p* = 0.033) when compared to mice treated with placebo (39.9 ± 1.96). Leptin did not affect PaCO_2_ in lean male mice (placebo 39.89 ± 4.34; leptin 40.95 ± 2.09) or lean female mice (placebo 37.41 ± 3.70; leptin 40.84 ± 2.14) (obese male *n* = 16; obese female *n* = 14; lean male *n* = 12; obese female *n* = 15). Statistical analyses were conducted, as shown in Figure 9.

**FIGURE 11:**
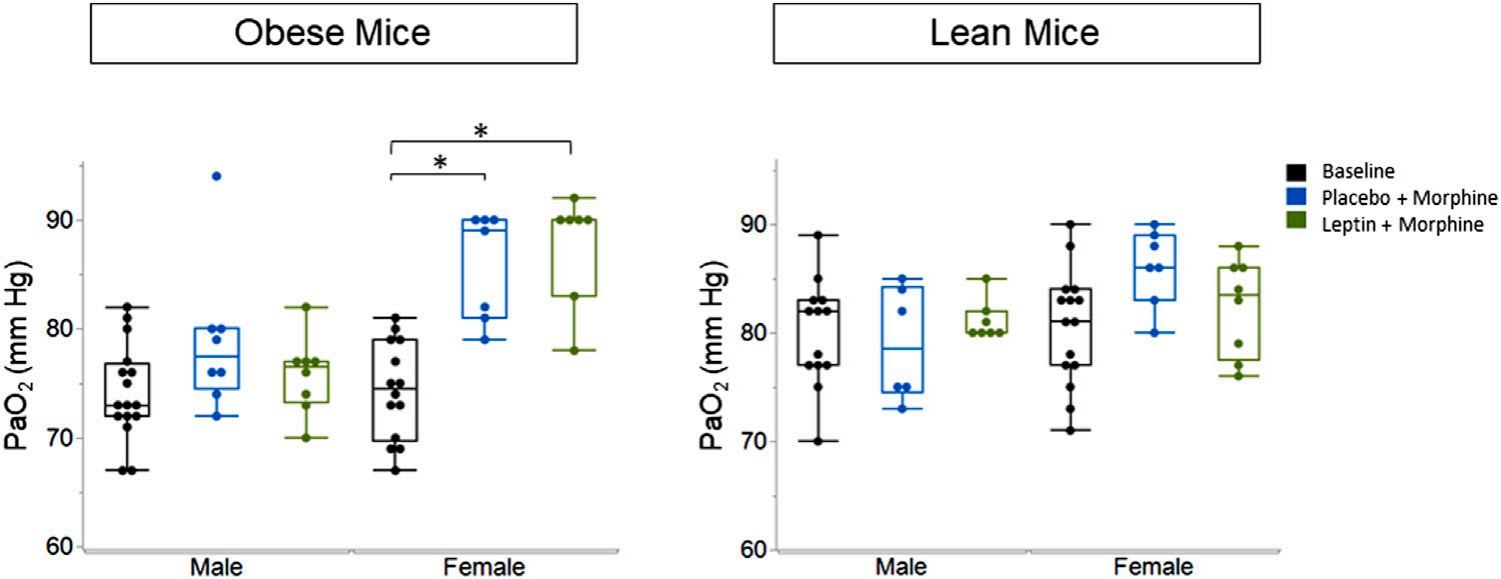
Blood gas: partial pressure of oxygen (PaO_2_) in obese and lean mice, following intraperitoneal injection of morphine. There was no significant difference in PaO_2_ between placebo and leptin groups with respect to sex or weight. Obese males (placebo 75.13 ± 4.8; leptin 73.25 ± 3.99). Lean male placebo (86.0 ± 3.51); lean male leptin (82.38 ± 4.5). Obese female placebo (85.86 ± 4.95); obese female leptin (87.57 ± 5.09); lean female placebo (79.0 ± 5.25); lean female leptin (81.14 ± 1.86) (obese male *n* = 16; obese female *n* = 14; lean male *n* = 12; obese female *n* = 15). Statistical analyses were conducted, as shown in Figure 9.

IN leptin stimulates both breathing and metabolism. Therefore, we chose to complement our respiratory data with measures of arterial blood gas, the gold standard for the acid–base state. At baseline, all four groups of mice (Table 1) showed similar pH and PaCO_2_ values. In DIO mice of both sexes, the delivery of morphine bolus resulted in a significant reduction in arterial pH and an increase in PaCO_2_ (Figures 9, 10). However, administration of IN leptin maintained pH, which remained significantly higher when compared to the placebo (*p* = 0.02). In lean mice, pH remained stable across treatment conditions with no appreciable differences between groups (*p* = 0.87). In summary, morphine induces respiratory acidosis in obese male and female mice, as well as lean male and female mice. However, IN leptin prevented acute respiratory acidosis in morphine-induced OIRD only in male DIO mice. Compared to the placebo, IN leptin increased pH in this group from 7.37 to 7.41 (*p* = 0.02), decreasing PaCO_2_ (from 39.9 to 36.3). In contrast, in DIO female mice, IN leptin decreased pH and increased PaCO_2_. In lean male mice, neither morphine nor leptin appeared to have an effect on the acid–base state. In lean female mice, morphine induced respiratory acidosis by increasing PaCO_2_, whereas IN leptin was ineffective. In DIO female mice, there was inexplicable increase in PaO_2_ by morphine (Figure 11), while in other groups of mice, PaO_2_ was unchanged. IN leptin did not affect PaO_2_ in any group of mice. Thus, IN leptin was effective in preventing the development of respiratory acidosis in DIO male mice. However, it was not helpful in lean mice of both sexes and appeared to exacerbate acid–base disturbances in DIO female mice.

**TABLE 1:** Mouse weights for blood gas experiments. Mean weights (SD) are shown at baseline (prior to femoral artery cannulation) for all experimental groups. (Obese male *n* = 16; obese female *n* = 14; lean male *n* = 12; obese female *n* = 15).

Sex	Group	Condition	Weight (g)
Male	Lean	Placebo	32.9 (±1.55)
		Leptin	33.1 (±1.56)
	Obese	Placebo	47.1 (±4.57)
		Leptin	48.2 (±4.54)
Female	Lean	Placebo	22.8 (±1.48)
		Leptin	23.5 (±1.64)
	Obese	Placebo	42.1 (±5.73)
		Leptin	42.4 (±5.38)

## 4 Discussion

The main finding of our study was that IN leptin effectively treated OIRD in morphine-tolerant DIO male mice without impacting analgesia. In contrast, IN leptin had no effect in lean mice of either sex or DIO female mice. The arterial blood gas data were consistent with ventilatory findings showing that IN leptin reversed morphine-induced respiratory acidosis only in DIO male mice but not in other mouse groups. Finally, a hypercapnic sensitivity study revealed that IN leptin rescued minute ventilation under hypercapnic conditions only in DIO male mice, which suggests that differential responses to IN leptin are attributable to different leptin sensitivities depending on sex and the obesity status.

Sex differences in OIRD susceptibility are insufficiently studied. One study reported significant sex and genetic effects on respiratory sensitivity to opioids in outbred mice (Bubier et al., 2020). Our data suggest that in the C57BL6/J strain, female mice have a significantly higher number of apneas in response to the same dose of morphine compared to males. In DIO male mice, IN leptin abolished morphine-induced decrease in minute ventilation in 8% CO_2_, while room air ventilation was not affected, which suggests that IN leptin alleviated OIRD by enhancing hypercapnic sensitivity. This stimulatory effect on breathing resulted in the resolution of respiratory acidosis, according to the blood gas data. Mechanisms by which leptin increases CO_2_ sensitivity are insufficiently understood. Leptin binds to the long isoform leptin receptor LEPR^b^ of CO_2_-sensing neurons, but localization of these neurons has not been established (Pho et al., 2020). Several laboratories localized respiratory effects of leptin to the nucleus of the solitary tract (NTS) (Inyushkin et al., 2009; Inyushkina et al., 2010; Do et al., 2020; Yu et al., 2021). However, chemogenetic stimulation of LEPR^b^ (+) NTS neurons did not treat hypoventilation in DIO mice (Amorim et al., 2021). We have shown that expression of LEPR^b^ in the dorsomedial hypothalamus (DMH) of *Lepr* ^*b*^ *-*deficient *db/db* mice restores respiratory responses to leptin (Pho et al., 2020), suggesting that DMH is a potential site of respiratory effects of leptin. Mechanisms by which LEPR^b^(+) respiratory neurons counteract OIRD are not clear. Nevertheless, our data suggest that there is a neural circuit between the LEPR^b^ (+) respiratory neurons and MOR (+) neurons implicated in OIRD (Bateman et al., 2021).

IN leptin is effective for OIRD treatment in opioid-naïve and opioid-tolerant DIO male mice but not in other groups. The arterial blood gas data and hypercapnic sensitivity data corroborated these findings. We propose that the differential effects of IN leptin in different weight and sex groups are due to different sensitivities to respiratory effects of leptin. We have previously shown that DIO male mice have high levels of circulating leptin, but they develop SDB and hypoventilation during sleep. IP and IV leptin injections fail to treat apneas, increase ventilation, and induce leptin receptor signaling in the brain in DIO mice (Fleury Curado et al., 2018; Berger et al., 2019; Freire et al., 2022). This leptin-resistant state is likely due to the limited permeability of the blood–brain barrier (BBB) to leptin and relative CNS leptin deficiency. IN leptin delivery circumvents BBB and delivers leptin to the brain centers, augmenting leptin receptor signaling and treating both SDB and OIRD (Berger et al., 2019; Freire et al., 2020). In contrast to DIO male mice, DIO female mice increase minute ventilation as they gain weight, showing no evidence of hypoventilation or increased apneas during sleep (Kim et al., 2023). Lean male mice show higher hypercapnic sensitivity than DIO male mice and have no SDB (Kim et al., 2022). Resistance to respiratory effects of leptin at the BBB results in CNS leptin deficiency. The respiratory effect of leptin in DIO male mice is saturable, and leptin at higher does (2.4 and 4.8 mg/kg) does not provide additional benefits, probably because of the high occupancy of the leptin receptor and the resolution of CNS leptin deficiency at 0.6 and 1.2 mg/kg. DIO female and lean mice of both sexes are not leptin-resistant and do not have CNS leptin deficiency. Therefore, exogenous leptin is not beneficial in these groups. Taken together, it is suggested that resistance to respiratory effects of leptin at the BBB level is specific for DIO male mice and does not occur in DIO female mice or in lean mice. Future studies with LEPR^b^ blockers in DIO female and lean mice can provide definitive evidence that endogenous leptin protects these animals from OIRD. Overall, our study suggests that IN leptin delivery is effective only in DIO male mice because it circumvents leptin resistance, which is absent in other animal groups.

We have previously shown that IN leptin is effective for OIRD treatment in opioid-naïve mice. However, in real-life situations, the majority of OIRD cases occur in patients who are chronically on opioids, either due to chronic pain or due to opioid use disorder. These patients develop opioid tolerance, requiring escalation of the opioid dose which may result in OIRD. We have modeled the opioid-tolerant state with the dose escalation by morphine boluses and shown that IN leptin is effective for OIRD prevention in opioid-tolerant mice at the same dose as it was in the previous study in opioid-naïve animals. Our new findings suggest that effects of IN leptin are similar in the opioid-naïve and opioid-tolerant states.

Our study had several limitations. First, morphine dose titration has been performed only in male mice. We found that IP morphine at 10 mg/kg induced a high number of apneas in females and, therefore, considered the titration unnecessary (Figure 5). Second, we were unable to perform blood gas measures in morphine-tolerant mice. Following morphine pump placement, mice had severe akathisia, which resulted in twisting and dislodging of the femoral arterial catheters, which prevented consistent blood sampling. Therefore, ABG experiments were conducted in morphine-naïve animals. Third, there was a discrepancy between the lack of suppression of minute ventilation in DIO female mice, according to the plethysmography measurements and blood gas data showing profound respiratory acidosis with decreases in pH and increases in PaCO_2_. One possibility is that DIO female mice have increased CO_2_ production, which would increase PaCO_2_ without a simultaneous increase in minute ventilation. Opioids induce excessive motor activity via MOR (Acevedo-Canabal et al., 2023; C. R; RETHY et al., 1971; Varshneya et al., 2019). Respiratory analysis required exclusion of periods of excessive locomotion with artifacts. We were unable to compare the effect of leptin on morphine-induced akathisia in this study. Our study illustrates the importance of the arterial blood gas analysis for comprehensive assessment of OIRD, which allows for avoidance of these artifacts. Fourth, although our current data and previous reports (Freire et al., 2020) suggest that selective efficacy of IN leptin in DIO male mice is due to leptin resistance, ‘loss of function’ studies with LEPR^b^ blockers in DIO female mice and lean mice of both sexes will provide definitive support to this hypothesis. Fifth, although we have shown that leptin treats OIRD by increasing hypercapnic sensitivity, mechanisms by which leptin counteracts OIRD remain elusive and should be a subject of future investigations.

As previously mentioned, obese females demonstrated preserved ventilatory function under both normoxic and hypercapnic challenge conditions, regardless of intervention. However, this preservation of function was not reflected in ABG data. One reason for this discrepancy may be that ABG data were collected in morphine-naïve animals, as opposed to morphine-tolerant animals. Chronic vs. acute administration of morphine may act differently upon obese female mice. Alternatively, a difference in the metabolic rate may impact results. Respiratory data were collected during periods of rest, while animals were often more active and moving about their home cages during blood gas experiments. Further exploration of these results and underlying mechanisms is warranted.

## 5 Conclusion and implications

IN leptin treats OIRD in DIO male mice, but it is ineffective in DIO female mice and lean mice of both sexes. Given the IN leptin selective efficacy in only one experimental group, these pre-clinical data should be carefully considered to decide on future clinical trials.

## Data Availability

The raw data supporting the conclusion of this article will be made available by the authors, without undue reservation.
